# Paving Paradise: The Peril of Impervious Surfaces

**DOI:** 10.1289/ehp.113-a456

**Published:** 2005-07

**Authors:** Lance Frazer

Paved surfaces are quite possibly the most ubiquitous structures created by humans. In the United States alone, pavements and other impervious surfaces cover more than 43,000 square miles—an area nearly the size of Ohio—according to research published in the 15 June 2004 issue of *Eos*, the newsletter of the American Geophysical Union. Bruce Ferguson, director of the University of Georgia School of Environmental Design and author of the 2005 book *Porous Pavements*, says that a quarter of a million U.S. acres are either paved or repaved every year. Impervious surfaces can be concrete or asphalt, they can be roofs or parking lots, but they all have at least one thing in common—water runs off of them, not through them. And with that runoff comes a host of problems.

Globally, it is a little more difficult to judge the square mileage of impervious surfaces. “We can extrapolate from the United States to a degree,” says Ferguson, “but there are too many variables to judge accurately.” The United States has a lot of automobiles, and compared to many other countries, Americans tend to build more (and wider) roads, more (and bigger) parking lots, more (and more expansive) shopping centers, and larger houses (with accompanying larger roofs). He says, “The United States might be on a par with Europe, but we’d be very different from India, for example, or any country where large numbers of the populace live in smaller, scattered villages, mostly without paved roads, parking lots, and the like.”

According to the nonprofit Center for Watershed Protection, as much as 65% of the total impervious cover over America’s landscape consists of streets, parking lots, and driveways—what center staff refer to as “habitat for cars.” Says Roger Bannerman, a researcher with the State of Wisconsin Department of Natural Resources: “You see some truly insane things in this country. I’ve seen subdivisions with streets that are thirty to forty feet wide. That’s as wide as a two-lane highway. Most developers are going back to a twenty-five- to twenty-eight-foot width, but you can still see these huge streets.”

Upon these automotive habitats fall a variety of substances, and thereby hangs the rest of the tale. Impervious surfaces collect particulate matter from the atmosphere, nitrogen oxides from car exhaust, rubber particles from tires, debris from brake systems, phosphates from residential and agricultural fertilizers, and dozens of other pollutants. “On a parking lot, for example, we have demonstrated buildups of hydrocarbons, bacterial contamination, metals from wearing brake linings, and spilled antifreeze,” says Ferguson.

On a road of open-graded aggregate (stone), much of that material would seep down into the pavement and soil, and the community of microorganisms living there would begin a rapid breakdown process. But pollutants can’t penetrate an impervious surface, and the rapid flow of rainwater off of impervious surfaces means these pollutants end up in the water. “So then,” says Ferguson, “not only do you have too much water, all moving too fast, you have polluted water that kills fish and makes water unfit for drinking or recreation.”

## When Water Has Nowhere to Go

Areas across the country are being impacted by the growth in coverage by impervious surfaces. In Maryland, for example, when watershed imperviousness exceeds 25%, only hardier reptiles and amphibians can thrive, while more pollution-sensitive species are eliminated, according to a 1999 Maryland Department of Natural Resources report titled *From the Mountains to the Sea*. Watershed imperviousness exceeding 15% results in streams that are impossible to rate “good,” states the report, and even 2% imperviousness can affect pollution-sensitive brook trout.

The 1.1-million-acre Chesapeake Bay watershed, one of the most diverse and delicate ecosystems in the world, is now being impacted by the 400,000 acres of impervious surfaces in Maryland. The Great Lakes, the streams and rivers of the Pacific Northwest, the Everglades of Florida—all are being impacted in one or more ways by runoff from streets, parking lots, and rooftops.

Bannerman has spent the last 30 years studying stream flow and the effect of urbanization on watersheds, including the depletion of groundwater reserves. “Not allowing the rainfall to infiltrate back into the aquifer is a very serious issue,” he says. “If that happens, you lose the base flow [the portion of water derived from underground sources] for streams, and you lose the wetlands fed by springs. It’s a complete disruption of the hydrologic cycle.”

Bannerman cites the example of Lake Wingra, a 1.3-square-kilometer lake in Madison. “A hundred years ago,” he says, “this lake was fed by around thirty-five separate springs. But today, because the lake is now almost entirely surrounded by urban areas, there are only four streams feeding the lake. Local organizations have gotten active in trying to restore the lake’s water quality, but it’s not the same lake it was a hundred years ago.” Lake Wingra now suffers from algal blooms caused by overfertilization, beach closures due to bacterial contamination, turbidity, and drying of surrounding wetlands.

Bruce Wilson, a research scientist with the Minnesota Pollution Control Division, is midway through a satellite survey of impervious surface area in that state. What Wilson has seen thus far is enough to cause significant concern about the state’s growth and development, and the impact of impervious surfaces on the water system.

“Impervious surfaces are impacting the lakes and streams on a number of fronts,” he says. “Velocity of runoff is a big one. Water runs off of these surfaces so rapidly, it creates mini-tsunamis that can cause serious, even irreparable, harm to the stream ecosystem. . . . And of course, the ability to recharge the groundwater system is being impacted. If you get into a twenty- to thirty-percent drop in infiltration [into the aquifer], which means a loss of base flow, the impact on streams being fed by surface water gets magnified still further.”

Another big problem for urban areas is the flash flooding that can occur when heavy rains fall over a city, according to hydrometeorologist Matt Kelsch, an authority on urban flash flooding with The University Corporation for Atmospheric Research in Boulder. Since runoff from an acre of pavement is about 10–20 times greater than the runoff from an acre of grass, Kelsch says impervious surfaces can quickly trigger devastating floods that can produce a host of their own environmental health hazards.

“In urban areas, anywhere from thirty to forty percent of the rainfall runs right into whatever stream is in the area, and in heavily urbanized areas it can be more than fifty percent,” he explains (by comparison, he says, the amount of runoff in subsaturated woodlands is often less than 5%). “If the water overflows the stream banks, it’s going to seek the path of least resistance. In most cases, that’s going to be the roadways.”

In many desert areas, Kelsch says, engineers take advantage of the natural topography, building houses at higher elevations and installing roads that lead up to residential areas. What this does is make the roads far more dangerous. More than 50% of the fatalities in flash floods occur on roads, according to Kelsch.

Floods are often given numerical designations such as “hundred year flood,” meaning such a flood happens once every 100 years (or has a 1% chance of occurring in any given year). The Federal Emergency Management Agency maintains a national list of flood zones and maps of impacted areas. The problem, says Kelsch, is that we’ve changed the playing field. “A couple of factors come into play,” he says. “First, this is still a pretty new country, so most places haven’t been developed long enough to know about the historical risk of a devastating flood. Secondly, when we urbanize an area, we alter the historical frequency of these events. The more we develop an area, the more rainfall we put as runoff directly into streams that have evolved to handle only a fraction of that runoff, and the more that happens, the greater the likelihood of a catastrophic flood.” Several such floods hit New Orleans in the 1980s, and three hit St. Paul–Minneapolis between 1990 and 2001.

## Heat Islands and the Stream

Wilson is also studying the “heat island” impact on Minnesota’s trout streams, an impact he says evidence and experience suggest is significant. Impervious surfaces, particularly roads and parking lots, are generally dark, and thus heat-absorbing, so they heat the rainwater as it hits. A sudden thunderstorm striking a parking lot that has been sitting in hot sunshine (where surface temperatures of 120°F are not unheard of) can easily yield a 10°F increase in rainfall temperature. And that heated water isn’t coming off just one parking lot or one street, but more likely several, all adding heated water to a stream or river.

Many aquatic organisms, at different stages of their lives, are vulnerable to even small increases in water temperature. “I’ve seen trout streams in Wisconsin and elsewhere in the Midwest lose whole populations because of—at least in part—the rise in temperature caused by runoff from impervious surfaces,” Wilson says. “Increased temperature also decreases the water’s ability to hold oxygen, which has a further detrimental effect on the aquatic life.” Warm temperatures can cause a variety of problems for fish, including decreased egg survival, retarded growth of fry and smolt, increased susceptibility to disease, and decreased ability of young fish to compete for food and to avoid predation. Especially affected are species that require cold water throughout most stages of their lives, such as trout and salmon.

Eventually, given no additional changes, the temperatures would drop, but in the interim the impact on wildlife could be serious. Oregon is one state that is examining the science of water temperature effects on stream life. Oregon standards for optimal salmon and trout rearing and migration call for water temperatures of 64.4°F. According to a 2004 report by the Oregon Independent Multidisciplinary Science Team, which advises the state government on scientific matters related to the Oregon salmon and watershed management, studies have shown that adult salmon begin to die off at temperatures of 69.8–71.6°F, and some species of trout at slightly higher temperatures. Although young salmon can survive slightly higher temperatures, the impact on their growth and survival rate is well documented.

## Impact of Building Materials

Not yet as well documented is the impact of pollutants released into stormwater runoff by building and paving materials themselves. Asphalt is one concern, as it contains coal tar pitch, a recognized human carcinogen, as well as polycyclic aromatic hydrocarbons (PAHs) including benzo[*a*]pyrene, another carcinogen. Another potential source of pollution is wood used for utility poles, play structures, and other structures that has been treated with chromated copper arsenate (CCA; a substance now being phased out due to health concerns), pentachlorophenol, or creosote. According to a paper presented at the 2004 Annual Water Resources Conference by Melinda Lalor, a professor of environmental engineering at the University of Alabama at Birmingham, in 1987 the United States alone produced some 11.9 million cubic meters of CCA-treated wood, 1.4 million cubic meters of pentachlorophenol-treated wood, and 2.8 million cubic meters of creosote-treated wood. And structures, once built from such materials, are intended to last a long time. The health risks of arsenic and chromium are well known, and while copper is not generally a human health risk, low concentrations of certain ionic forms of this metal are toxic to marine flora and fauna.

“In general,“ says Lalor, “pollutant level tends to vary depending upon the age of the material, and the harshness of the environment to which it is exposed. As material ages and is exposed to high levels of sunlight, temperature extremes, chemicals in the environment such as salt from roads, and so on, leaching out will increase.”

If the pollutant source is a coating, then pollution levels decrease with age, but can still have a significant impact, she says. “If you look at the asphalt used in a parking lot, the top coat is quite toxic. So if you have a heavy rain [soon] after the parking lot goes in, it’s not unusual to see fish kills downstream.”

Lalor cites research published in volume 35, issue 9 (1997) of *Water Science and Technology* showing that stormwater from roofs and streets contributed 50–80% of the cadmium, copper, lead, and zinc measured in Swiss combined sewer system flows. Polyester roofing materials shed the highest concentrations of metals, followed by tile roofs, then flat gravel roofs. The Swiss researchers also found PAHs and organic halogens in the roof runoff.

The chemicals released can have a significant impact on environmental and potentially human health. “Some materials, such as metals, are especially toxic to fauna at various stages of their life cycle,” says Lalor, “while some organics, particularly petroleum-based organics, can function as pseudoestrogens. So while they may not cause death, they can trigger a significant disruption in the physiology of the organisms exposed to these pollutants.”

According to Lalor, although there are mandated tests for urban stormwater discharge, there are currently no tests mandated for building materials to determine their potential for toxics release. “If a community wants to develop around their drinking water source, they should know about release potential from building materials so they can carefully select those with which they build,” she says. “We don’t yet have the science to support it, but it would be a positive step to be able to go to a builder and say, ‘Look, here’s a list of twelve building material alternatives that would be most environmentally benign for this site and these conditions.’”

Lalor says New Zealand has been the leader in this sort of study, and that nation is preparing to put regulations in place regarding building materials and environmental impact. But such studies haven’t been elevated to a high enough priority in the United States to build the science we need for setting new policies. She adds, “We need to address the entire life cycle of building materials, from what goes into their creation, to the impact of construction on the environment, to the impact of whatever might leach out during their lifetime, to the end-of-life disposal issues.”

## The Promise of Porous Pavements

Despite the overwhelming body of evidence supporting the negative relationship between impervious surfaces and the environment, no one would seriously suggest that we stop paving streets or building parking lots. What, then, are the options?

According to Ferguson, there are nine different families of porous pavement materials. Some of these materials are already well known in the United States; they include open-jointed pavers that can be filled with turf or aggregate, “soft” paving materials such as wood mulch and crushed shell, and traditional decking.

Other families include porous concretes and asphalts being developed by engineers and landscape architects. Ferguson says these materials use the same components and manufacturing processes as conventional impervious materials, “and as a general rule, carry the same health and environmental issues. . . . Same chemicals, same energy costs to manufacture, but far different benefits in its use.” These new formulations still provide solid, safe surfaces for foot and vehicle traffic, but also allow rainwater to percolate down into subsurface soils.

The porosity of porous asphalt is achieved simply by using a lower concentration of fine aggregate than in traditional asphalt; it can be mixed at a conventional asphalt plant. Under the porous asphalt coating is a bed of clean aggregate. Importantly, this aggregate is all of the same size, which maximizes the void spaces between the rocks, allowing water to filter through. A layer of geotextile fabric beneath this bed lets water drain into the soil and keeps soil particles from moving up into the stone.

Porous asphalt was actually developed more than 30 years ago, according to Ferguson, but it didn’t pan out at that time. Part of the problem, he believes, was—and continues to be—the low level of federally funded research. “Back in the early eighties, when porous pavement was new, the Environmental Protection Agency [EPA] was really interested, especially in porous asphalt,” he says. “But one of the problems with porous asphalt back then was that on a hot day, the binder softened and migrated down to a cooler layer. That released the surface aggregate and clogged the lower layer.” According to Ferguson, the EPA became discouraged and discontinued studies.

Since then, however, porous asphalt technology has been improved by French, Belgian, and Irish researchers, Ferguson says. During the late 1980s and early 1990s, they discovered that adding polymer fibers and liquid polymers to the asphalt prevented the binder from draining down through the aggregate. “Today, even though [porous asphalt] started out here, what we’re using has been imported back from Europe,” he says.

Ferguson says porous pavement constitutes only a minute fraction of all the paving done each year in the United States. “However,” he continues, “the rate of growth of porous paving, on a percentage basis, is very high, primarily because of public concern about and legal requirements for urban stormwater management. This growth is happening both in the big asphalt and concrete industries, and in the smaller industries that supply competing materials such as concrete blocks and plastic geocells.”

One argument against pervious surfaces in high-traffic areas is that they’re not as durable as their impervious ancestors. That, says Ferguson, is simply not true. “I’ve seen pervious pavement in good shape in places like Minnesota and Alaska, where you have tremendous climatic extremes,” he says. “In Georgia and Oregon, it’s now routine to resurface highways by putting a layer of pervious asphalt over the impervious surface below. That way, water drains laterally below the surface, giving you better traction and visibility.” Although the major advantage to this practice is highway safety, rather than reinfiltration of the water into the groundwater, it still allows for more water to return to the groundwater table than would be the case with an impervious surface, where it merely evaporates back into the atmosphere.

Some pervious surfaces have the additional benefit of allowing pollutants to come into contact with microbes beneath the surface. According to Ferguson, these naturally occurring microbial communities thrive on the large surface area of the pervious pavements’ internal pores and break down contaminants (particularly petroleum by-products) before they can leach down into the water supply.

“Coventry University scientists did a study recently, where they applied oil to a lab mockup of a porous road surface,” he says. “They dumped far more used oil on the surface than you’d ever find accumulating on a parking lot, and none of it reached the soil layer below”—instead, microbes digested it all. The Coventry team, led by Christopher J. Pratt, published an overview of their work in the November 2004 issue of the *Quarterly Journal of Engineering Geology and Hydrogeology*.

## Other Ways of Controlling Runoff

Approaches to dealing with the spread of impervious surfaces go beyond changing the building material itself. Kelsch says a return to more reasonable street width is one measure, and many communities are increasing their number of green areas as a means of allowing rainfall to infiltrate back into the ground.

For urban areas with nearby lakes, Bannerman says construction of “rain gardens” is becoming a popular method that homeowners and businesses can use to help control stormwater runoff. Such gardens are designed with dips in the center to capture water, which then can slowly filter into the ground rather than run off into the storm sewer. Ideally these gardens are situated next to a hard surface such as a sidewalk or driveway, and are planted with hardy native species that can thrive without chemical fertilizers or pesticides.

Ponding basins like those used in Fresno are another option. This city of just over half a million in Southern California’s San Joaquin Valley gets less than 12 inches of rain annually and draws most of its water from underground aquifers and the nearby Kings and San Joaquin rivers. Beginning in the late 1960s, the city started constructing several ponding basins—large basins where stormwater can settle, then drain down through the soil. Water systems manager Lon Martin says the city had two goals in establishing these ponding basins: “First was to keep stormwater runoff from flooding the city and from going into the rivers, potentially causing water quality problems. Secondly, the city has begun a program of intentional aquifer recharging.”

To date, he says, the city has connected nearly 80 of the possible 150 ponding basins to its groundwater recharge system. Recharge from stormwater is one part of the equation, but the city also takes its May–October water allotment from the two rivers, diverts the water to these basins, and then allows gravity to pull the water down through the sandy loam soil into the aquifer.

Green roofs, another method of controlling rainwater runoff, are just what the name implies: roofs planted with all types of vegetation. Also known as “eco-roofs,” these surfaces can be either extensive (lighter in weight, relying on a few inches of soil and using plants like herbs, grasses, and wild-flowers) or intensive (much heavier, with a 12-inch soil depth that can accommodate trees and shrubs). According to the nonprofit Earth Pledge Foundation, green roofs can absorb nearly 75% of the rainfall that lands on them, and they can also reduce the urban heat island effect.

Green roofs perform several roles, one of which is water harvesting, or basically catching rainwater for use elsewhere. “This water is cleaner than that off the pavement,” Ferguson says. “[Water harvesting] is now being practiced in areas where water is less available, such as the Southwest or the Pacific Northwest, with their dry summers. . . . [It] can be a valuable tool in areas where water is scarce.”

In Germany, approximately 10% of the buildings have green roofs, and the city of Tokyo recently mandated that usable rooftop space of greater than 1,000 square meters atop new buildings must be 20% green. Green roofs are also found in North American cities including Chicago, Toronto, and Portland, Oregon.

## Beyond Imperviousness

Recognizing the environmental health threat of impervious surfaces as well as other point sources of pollution, the EPA established a stormwater permitting program under the National Pollutant Discharge Elimination System. Phase I of the stormwater program, promulgated in 1990, required permits for separate stormwater systems serving communities of 100,000 or more people, and for stormwater discharges associated with industrial and construction activity involving at least five acres. Phase II, promulgated in 1999, addressed remaining issues and urban areas of fewer than 100,000 people, as well as smaller construction sites and retail, commercial, and residential activities.

But further change will require a shift in how we think about runoff. Bannerman says, “What we’ve begun to do, and must continue to do, is to get away from the idea that rain is wastewater—something to get rid of, to pass along to our neighbors downstream. We need to keep it where it falls, and the way to keep it is to get it back into the ground.”

For flash floods, Kelsch says, “there is no solution. Flooding is going to happen, in spite of everything we can do. What we need to do is what we can to lessen the impact of the inevitable. That means building out of flood plains, and increasing the amount of rainwater we send back into the aquifers while decreasing the amount we discharge into streams.” Building design and use of permeable paving materials will help, he says, but we need to realize these aren’t total solutions. Further, he adds, “If we get stuck in the mindset that we have to have a solution, we may not do anything. And that will make the problem still worse.”

## Figures and Tables

**Figure:**
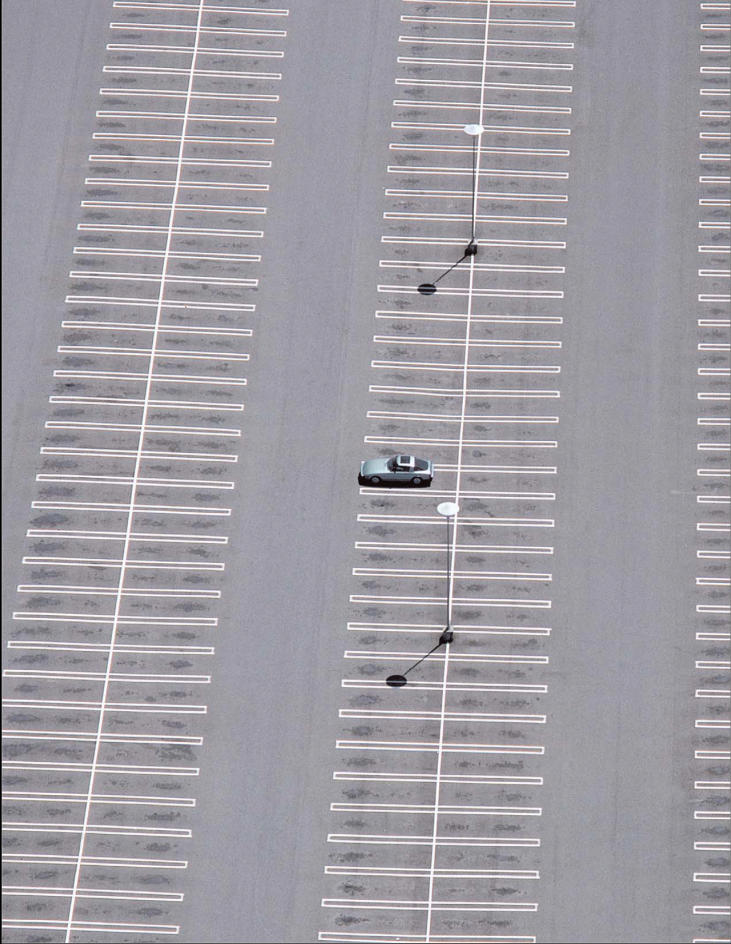


**Figure:**
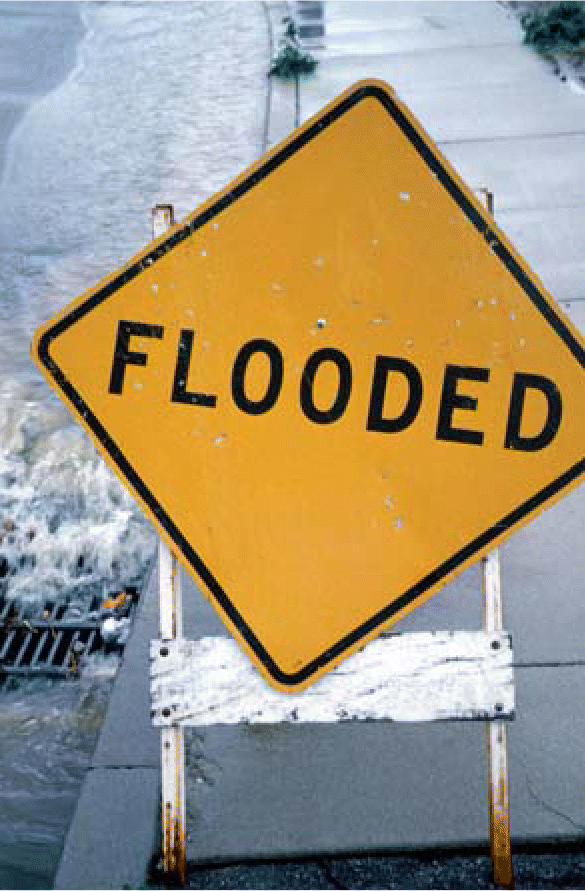


**Figure:**
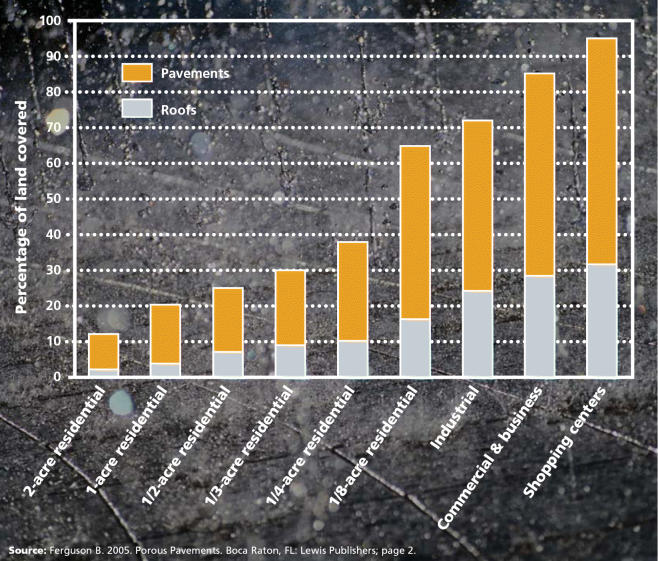
Impervious Cover of Various Land Uses

**Figure:**
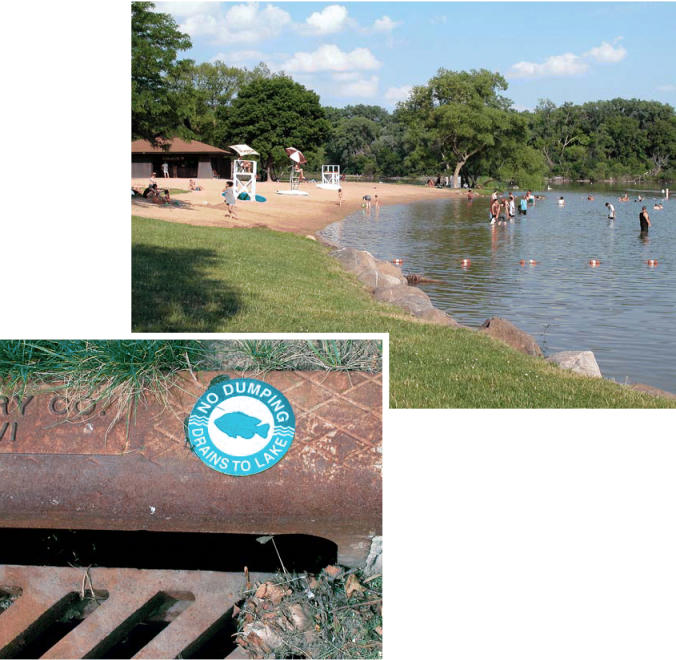
**Impervious to change?** Despite community efforts, Wisconsin’s Lake Wingra still suffers the effects of its urban surroundings including algal blooms, bacterial contamination, and turbidity.

**Figure:**
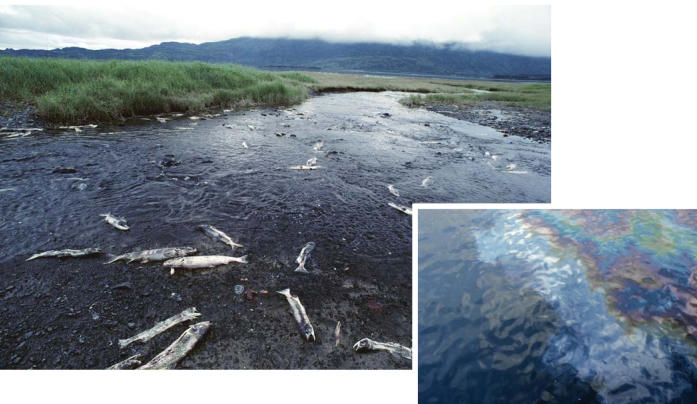
**Awash in toxicants.** Chemicals used in paved surfaces can be toxic to fish, wildlife, and possibly humans.

**Figure:**
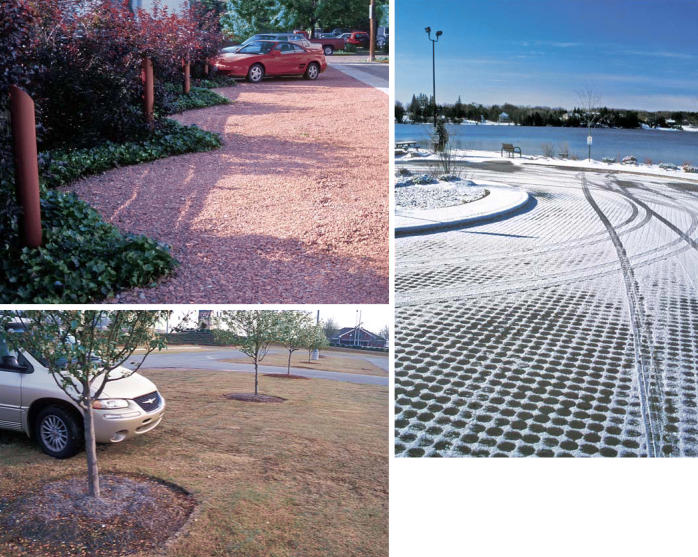
**Breaking through barriers.** Porous pavements come in many forms. Parking spaces in Columbus, Ohio (top left) are made of recycled clay aggregate. Shoppers at the Mall of Georgia, the largest mall in the U.S. Southeast, can park in a turf overflow lot (bottom left). The spaces between open-jointed pavers at Ontario’s Sunset Beach Park lakefront access lot (above) admit water and prevent pollution of Lake Wilcox.

**Figure:**
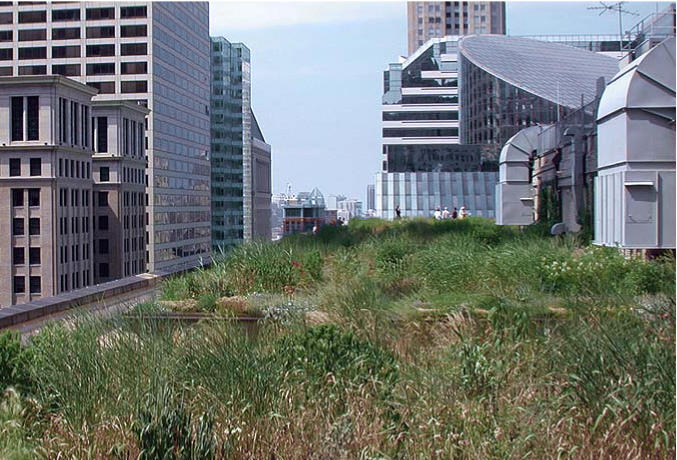
**On top of the problem.** The green roof atop Chicago City Hall contains more than 100 plant species that absorb stormwater and reduce the ambient air temperature by as much as 7–8ºF compared to a nearby tar roof.

